# How Air Temperature and Solar Radiation Impact Life History Traits in a Wild Insect

**DOI:** 10.1002/ece3.71135

**Published:** 2025-03-13

**Authors:** Alexandra S. Gardner, Ilya M. D. Maclean, Rolando Rodríguez‐Muñoz, Alfredo F. Ojanguren, Tom Tregenza

**Affiliations:** ^1^ Environment and Sustainability Institute University of Exeter Cornwall UK; ^2^ Centre for Ecology and Conservation University of Exeter Cornwall UK; ^3^ Departamento de Biología de Organismos y Sistemas Universidad de Oviedo Oviedo Spain

**Keywords:** adult mass, air temperature, cloud cover, development time, ectotherm, local adaptation

## Abstract

Ectotherms are essential components of all ecosystems. They rely on external heat sources like air temperature and solar radiation to regulate their body temperature and optimise life history traits. Climate change, by altering air temperature and cloud cover, will likely impact these processes. To examine how air temperature and shade influence terrestrial insects, we reared nymphs of the field cricket (*Gryllus campestris*) at high (mean air temperature 13.4°C) and low (mean air temperature 9.6°C) sites in northern Spain, with partially shaded and unshaded treatments at each site. We tested for local adaptation to these climate variables by rearing nymphs from high and low altitude genetic lineages in all treatment combinations. Development time was significantly longer (on average 10 days) at low air temperature but was unaffected by a 40% increase in shade, suggesting crickets compensate for reduced sun exposure in shaded environments and may forgo some opportunities to gain energy from the sun in unshaded environments. Adult mass was affected by an interaction between shade and air temperature. At low air temperature, shaded crickets had higher mass (on average + 0.06 g) than unshaded crickets, whereas at high air temperature, shaded crickets had lower mass than unshaded crickets (on average − 0.08 g). This indicates that changes in cloud cover will impact insects differently in warmer and cooler parts of their range. We found no evidence for local adaptation in either development time or mass, suggesting these traits are not strongly differentiated between populations from high and low altitude environments. Our findings highlight the importance of considering both air temperature and solar radiation when predicting climate change impacts on insects. Shifts in temperature and cloud cover may have complex and region‐specific effects on these vital ecosystem components.

## Graphical abstract

Ectotherms are essential components of all ecosystems and rely on external heat to regulate their body temperature. We studied the effects of air temperature and shading on crickets reared at different altitudes and found that development time was influenced primarily by temperature, whereas mass at adulthood depended on the interaction between shading and temperature. These findings suggest that changes in temperature and cloud cover due to climate change may have complex, region‐specific effects on insect populations.

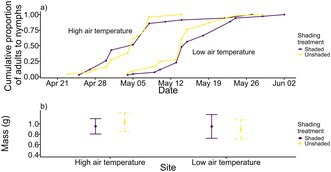

## 1 Introduction

Ectotherms make up the majority of terrestrial biodiversity (Wilson 1992). Any changes to their abundance and distribution due to climate change will have implications for biological systems globally. The thermal environment profoundly impacts ectotherms because they rely on external sources of heat to regulate their body temperature (Angilletta 2009). Body temperature directly affects many physiological processes, including digestion, movement, and growth (Buckley et al. 2001; Somero 2005). These physiological processes, in turn, influence essential traits such as growth rate, reproductive success, and survival, all of which contribute to an individual's overall performance and fitness. Ultimately, this means that the thermal environment shapes the distribution patterns of ectothermic species (Sunday et al. 2014).

Air temperature and solar radiation play a crucial role in determining the availability of suitable thermal environments. By basking in sunshine, ectotherms can raise their body temperature by more than 20°C above ambient air temperature (at 2 m above the ground) (Gardner, Maclean, et al. 2024). Many ectotherms bask to maximize radiative heat gain and maintain body temperatures that maximize physiological performance (Lahondère 2023). The combination of air temperature and solar radiation has been observed to explain the distribution and abundance of insects at various spatial scales (Bryant and Shreeve 2002; Kührt et al. 2006). Typically, areas with higher solar radiation and suitable air temperatures support greater insect activity and abundance, as these conditions facilitate metabolic processes, enhance reproductive success, and extend active periods. Conversely, regions with low solar radiation or suboptimal air temperatures may see reduced insect abundance due to constraints on their ability to thermoregulate effectively (e.g., Kingsolver 1983).

These patterns underscore the critical interplay between environmental factors and insect ecology, influencing both local distributions and broader biogeographic trends (Bryant and Shreeve 2002; Kührt et al. 2006). However, few studies have investigated how the combination of air temperature and solar radiation interact to affect life history and fitness‐related traits, which ultimately drive these patterns (e.g., thermal stress: Buckley et al. 2013).

Air temperature and solar radiation are not identical in their potential to affect fitness. Solar radiation often varies enormously at small spatial scales through the availability of shade. Sun basking is a critical part of thermoregulation for many insect species, enabling them to reach optimal body temperature for physiological processes. Air temperature, on the other hand, often varies less over the same distance. This means that there is less opportunity for ectotherms to exploit this variation through thermoregulatory behaviour, although they may hide in burrows or other types of shelters to control their body temperature in relation to air temperature. Insects can move between sunlit and shaded areas to control their body temperature and enhance their fitness by improving efficiency in foraging, escaping predators, and finding mates (Golab et al. 2019). However, basking in sunshine often carries costs, including an increased risk of predation (Chabaud et al. 2023; Pitt 1999), and the potential for cellular damage from ultraviolet (UV) radiation (Mazza et al. 1999; Potter and Woods 2013). It is also worth noting that total reliance on solar radiation for effective thermoregulation is not always possible, due to the inherently transient nature of sunshine availability, both throughout the day and across the year.

Many studies have considered the ecological responses of ectotherms under future climate change scenarios, but much of this research has focused on changes to average air temperature (Gardner et al. 2019) or the altered timing of extreme events (e.g., Deutsch et al. 2008; but see Kearney et al. 2009). However, shifts in air temperature have interdependence with shifts in solar radiation through variation in cloudiness. In recent decades, warming temperatures have increased the air's moisture‐holding capacity, leading to greater cloud cover over much of the earth and a lowering of the diurnal temperature range (Dai et al. 1999; Cox et al. 2020). Clouds attenuate shortwave radiation and thus reduce the radiation absorbed by organisms. This can limit body temperature, even if air temperature is high (Gardner, Maclean, et al. 2024). The fact that temperature, radiation, and cloudiness are interrelated, and the historical focus on responses to these factors in isolation, prompted our research into how temperature and radiation interact to determine life history traits.

Growth and development rates of immature insects are two life history traits that depend on body temperature (Kingsolver 1989) and are major components of individual fitness. Larger body size is often considered favorable in terms of both ecological and sexual selection (Clutton‐Brock 1988). However, other selection pressures, such as resource limitation, predation risk, and heat stress, may favor smaller sizes (Blanckenhorn 2000). There is an inevitable trade‐off between development time and adult mass because insects could opt to emerge earlier as adults after a shorter period of growth (Hassall 2013). Building a larger body must also trade off against other fitness‐related traits because feeding involves exposure to risks such as predation (Bernays 1997), and resources used for growth could be used for other traits such as the immune system (Rantala and Roff 2005).

In this study, we measured the effects of air temperature and solar radiation on development time and mass at maturity in an annual insect, the field cricket, *Gryllus campestris*. Our experiments took place at one high and one low altitude site in Northern Spain. We took advantage of altitudinal differences in air temperature and manipulated solar radiation levels through shading treatments. There are abiotic and biotic differences, besides air temperature, between high and low altitude sites. For example, oxygen concentration is lower at higher altitude, which may affect respiration, and lower air density may reduce convective heat loss of insects (Dillon et al. 2006). However, these differences are only likely to be significant at altitudes higher than those we were using; convective heat loss at 70 and 1300 m above sea level is almost identical at the low wind speeds our crickets were exposed to (see Figure 3 in Dillon et al. (2006)). Air pressure is lower at higher altitude, but this is not expected to have important effects on flightless insects other than via temperature. By excluding predators by keeping crickets in large, turfed boxes covered with a thin wire mesh in both locations, we removed a major biotic difference that might be associated with altitude. As a result, we believe that air temperature is by far the most important environmental difference between neighbouring high and low altitude sites.

*G. campestris* , like many other insects, has a widespread distribution across altitudes (Panagiotopoulou et al. 2016). A broad altitudinal range may be possible because individuals express different phenotypes in different environments (Acasuso‐Rivero et al. 2019), or because geographically adapted phenotypes may evolve, that is, there is local adaptation. Understanding the potential for local adaptation (Williams 1966) allows us to be more general about responses across the species' range. For example, high altitudes are colder than lower altitudes, and crickets at higher altitudes may therefore have evolved mechanisms that enhance solar heat absorption, which will buffer them against negative impacts of shade. Understanding local adaptation will be important for predicting responses to climate change (Franks and Hoffmann 2012; Schilthuizen and Kellermann 2013) and potential future distributions.

In a previous laboratory study we investigated the growth rate of nymphs immediately after hatching in the spring, comparing the offspring of parents from high and low altitudes. We found that there was an interaction between rearing temperature and whether parents were from high or low altitude. Specifically, we found that newly hatched nymphs kept at a high temperature grew faster during the first few weeks of their lives when their parents were from low altitudes relative to those from high altitudes. However, we have no information about the growth that occurs post‐diapause, nor about the independent effects of air temperature and solar radiation and possible local adaptation of responses to these factors. In this study, we tested for possible genetic components associated with the impact of environmental conditions on post‐diapause growth and mass at maturity by including crickets from high and low altitude genetic lineages in all treatment groups.

## 2 Methods

### 2.1 Study Species

The field cricket *Gryllus campestris* is a typical annual temperate insect widely distributed in open grassland habitat from north Africa to northern Europe (Hochkirch et al. 2016). Eggs hatch from May to July, and nymphs remain active until the temperature drops too low in the autumn, at which point they enter a winter diapause as late‐stage instar nymphs. They emerge in early spring to resume foraging and growth and undergo one or two more nymphal moults before becoming adults from mid‐April to mid‐May. Both nymphs and adults use sun basking to raise their body temperature. Dark colouration may substantially reduce the basking time required to reach optimal body temperatures, as observed in other insects (e.g., grasshoppers, Forsman 2000). Previous modelling of *G. campestris* adults indicates that at an air temperature of 20°C, body temperature could be up to 25°C higher under high solar radiation (~600 W/m^2^) compared to low solar radiation (~100 W/m^2^) (Gardner, Maclean, et al. 2024).

### 2.2 Cricket Rearing and Breeding

**FIGURE 1:**
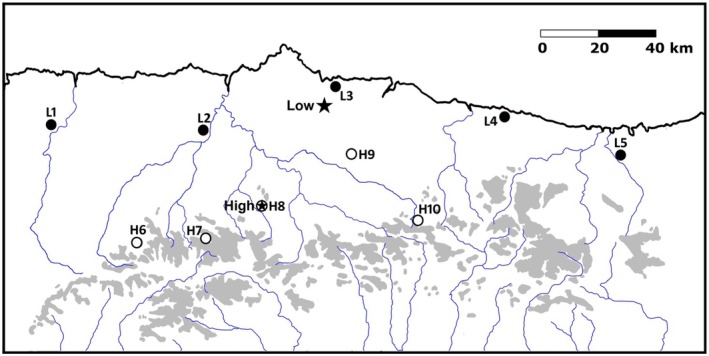
Locations of the collecting sites and experimental rearing sites. Collecting sites coded with L, are located < 170 m above sea level; the ones coded as H are located > 1100 m above sea level. Black stars indicate the low and high altitude experimental rearing sites. Grey colour represents areas > 1500 m above sea level and blue lines show the main rivers.

In spring 2021, we trapped crickets from ten populations in Asturias and Cantabria (Northern Spain) (Table 1, Figure 1). Five of the populations were located at altitudes below 120 m and five above 1120 m (Table 1).

**TABLE 1:** Location of cricket collection sites in 2021 in North Spain, with indications of capture date and the number of collected individuals; these are the parents of the crickets that we studied.

Population	Location	Coordinates	Altitude (m above sea level)	Date	FA	MA	FN	MN
L01	Trelles	43 29.247 N 6 43.671 W	85	20 May 2021	15	14	0	0
L02	Laneo	43 22.450 N 6 09.195 W	66	27 April 2021	+	+	+	+
L03	Trubia	43 30.059 N 5 45.083 W	75	26 April 2021	+	+	+	+
L04	Hontoria	43 27.081 N 4 54.859 W	46	01 May 2021	19	36	1	2
L05	Bielva	43 18.215 N 4 27.642 W	119	05 May 2021	19	30	0	0
H06	Brañas de Abajo	43 02.363 N 6 27.369 W	1240	08 May 2021	9	13	5	0
H07	Valle de Lago	43 04.096 N 6 11.180 W	1300	07 May 2021	0	0	7	7
H08	Llanuces	43 09.677 N 5 54.760 W	1118	05 May 2021	15	22	4	5
H09	Peña Mayor	43 16.498 N 5 30.206 W	1126	06 May 2021	7	5	7	5
H10	Tarna	43 06.299 N 5 13.369 W	1121	09 May 2021	4	32	0	1

*Note:* Population is the ID of each population. Coordinates are in UTM format. Date shows the date when the crickets were collected at each site. Low altitude sites were < 120 m above sea level (L01–L05), high altitude sites were > 1120 m above sea level (H06–H10).

Abbreviations: +, enough individuals captured, but with numbers not available; FA, female adults; FN, Female nymphs; MA, Male adults; MN, Male nymphs.

At the laboratory, we isolated up to 10 male and 10 female adults per population (depending on the numbers available) in 2 L plastic boxes. Each box had a window covered with a thin metal mesh for ventilation, a piece of egg cardboard for shelter, and food and water ad libitum. We stored the excess females (for those populations with more than 10 available) in groups of 4–5 using the same type of 2 L boxes, whereas males in excess remained grouped in the 20 L boxes used during capture. We placed the boxes on shelving within an open outdoor shed, where they remained in the shade, with ambient light and temperature. The only exception to this rearing protocol was population H07, for which we only captured nymphs. To promote adult emergence under natural conditions for population H07 nymphs, we placed them in 0.2m^2^ open‐top outdoor plastic boxes with a soil base and grass growing on it and sunflower seeds for additional food. Once they emerged as adults, H07 nymphs were isolated following the protocol described above for the rest of the populations. The males and females maintained in groups were only included in the protocol of isolation and mating (see below) when we had to replace any of the originally isolated individuals because of death or failure to mate and/or produce eggs.

To ensure that experimental females had mated with at least one male from mid‐May, we carried out mating trials with all the isolated females. At each mating trial, we put one pair of crickets from the same population into a 0.5 L plastic cup, with a piece of paper on the bottom to provide traction. We left the pair together in the cup and checked every few minutes or so over a period of 1–3 h to see if the female had a spermatophore attached under her ovipositor. We provided mated females with a 3 cm diameter Petri dish filled with wet sand. For females that did not mate in any given mating trial, we carried out further mating trials (maximum 3–5 trials) on subsequent days. After those trials, we discarded any female that did not mate in the lab and did not lay any eggs.

Every 1–3 days, we sieved the sand of each female's dish to collect any eggs and replaced it with a new dish with clean sand. We distributed the eggs of each sieved dish on a wet cotton‐wool pad placed in a Petri dish for incubation, trying to avoid clumps of eggs that could favor fungal growth. We left the dishes in the same room used to rear the laying females. At 25°C, incubation takes around 9 days; after 4–5 days, we checked incubation dishes daily to remove hatchlings and transfer them to a plastic box like those used for the isolated females. We reared all the hatchlings from consecutive laying events in the same box (i.e., only one hatchling box per female) and in the same room as laying females and incubation dishes.

**FIGURE 2:**
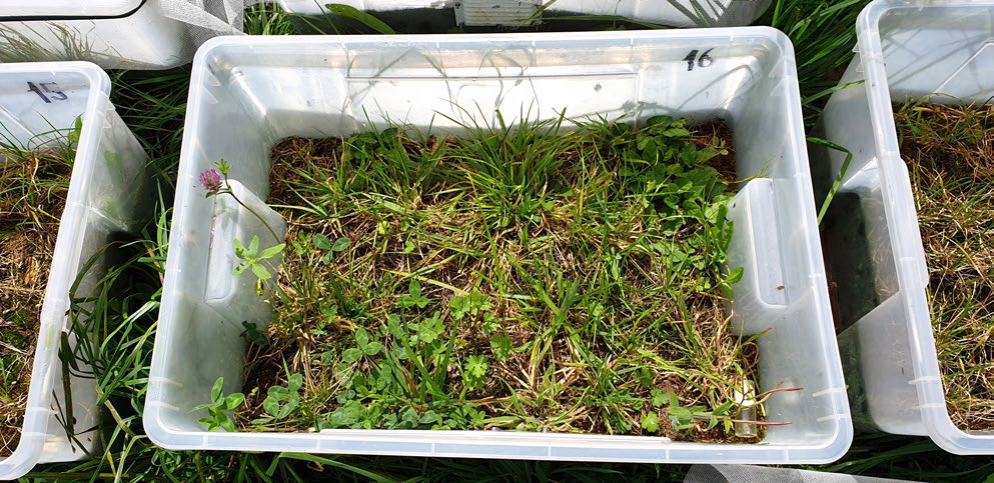
One of the 120 outdoor boxes into which nymphs were released.

Before releasing the nymphs, we determined the total number available in each family and divided it into four portions, one for each treatment: shaded at high altitude, unshaded at high altitude, unshaded at low altitude, and shaded at low altitude. Between 26/6/2021 and 20/7/2021, we released small nymphs into 120 outdoor translucent polypropylene boxes with an area of 0.2 m^2^, a depth of 28 cm, and a 14 cm layer of soil with natural grass growing on it (Figure 2). We aimed to release 30 nymphs per box at the start of the outdoor growing period, but there were a few families where we did not get enough or unwittingly added 1–2 extra individuals when dividing the families into four groups before being released (nymphs are tiny and very jumpy at this early stage). For the small number of females that did not produce 200 nymphs, we divided all the available nymphs into four groups and released each group into one of the boxes. Each box hosted nymphs from a single female. Sunflower seeds were provided ad libitum, and boxes were watered during periods of dry weather to prevent any competition for food or water and to ensure that access to nutrition did not differ between sites. Initial density was 25–32 nymphs in 110 boxes and 9–23 in 10 boxes. This minor variation in density was evenly distributed between the treatments of shaded/unshaded and identical between high and low altitude sites. This density variation is unlikely to have any significant effect on thermoregulatory behaviour, and because the shading treatment was evenly distributed, no bias was created.

**FIGURE 3:**
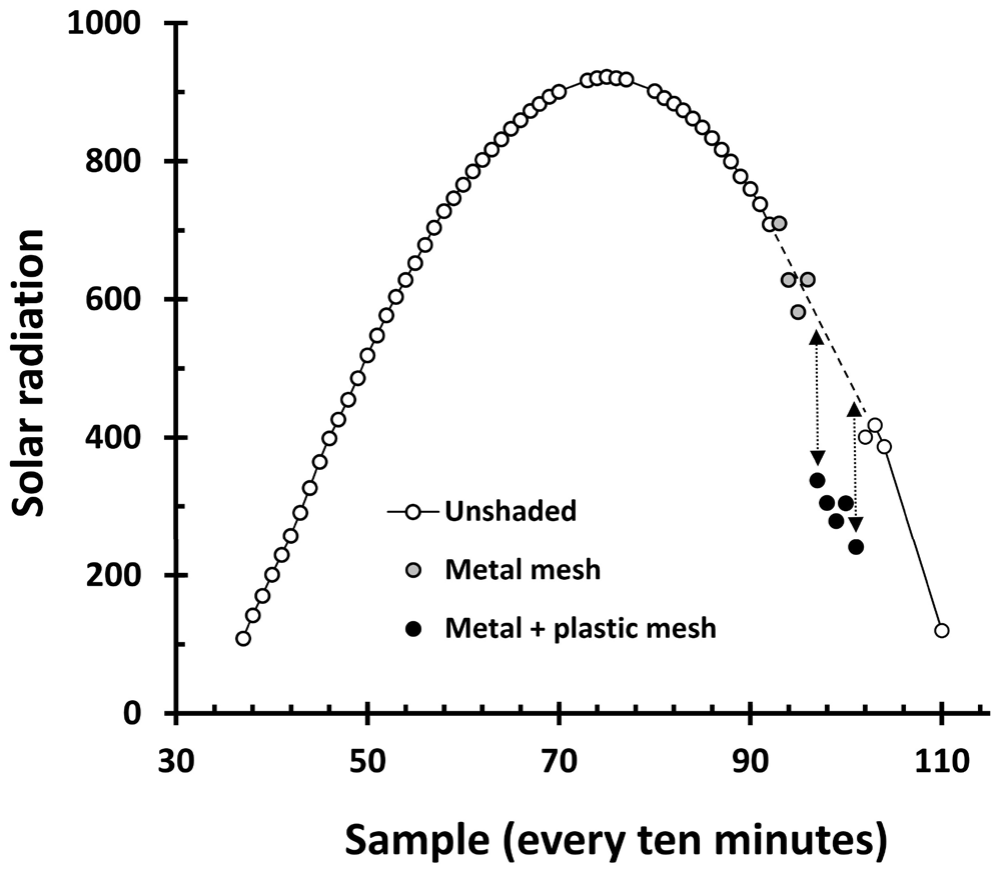
Solar radiation during a day of full sun measured at 1.5 m above the ground using Davis instruments, Vantage Pro2 Plus pyrometer. Grey dots show when a metal mesh was placed above the pyrometer which has a minimal impact on solar radiation. Black dots show a period where the plastic mesh treatment was applied; solar radiation can be seen to reduce by ~40% relative the expected value when unshaded.

Releasing took place in two batches, on the 26th of June and the 20th of July, for the earlier and later hatched nymphs, respectively. Boxes were initially covered with 0.5 mm steel mesh lids, as young nymphs are small and can climb up the inside of the box walls and escape. In mid‐October, when nymphs had grown larger and were subsequently unable to climb up the plastic walls, we replaced the thin mesh lids with wider 12 mm mesh lids. The lids prevented predation by birds but did not cast any appreciable shade (Figure 3).

### 2.3 Air Temperature and Shading Effects on Development Time and Adult Mass

**FIGURE 4:**
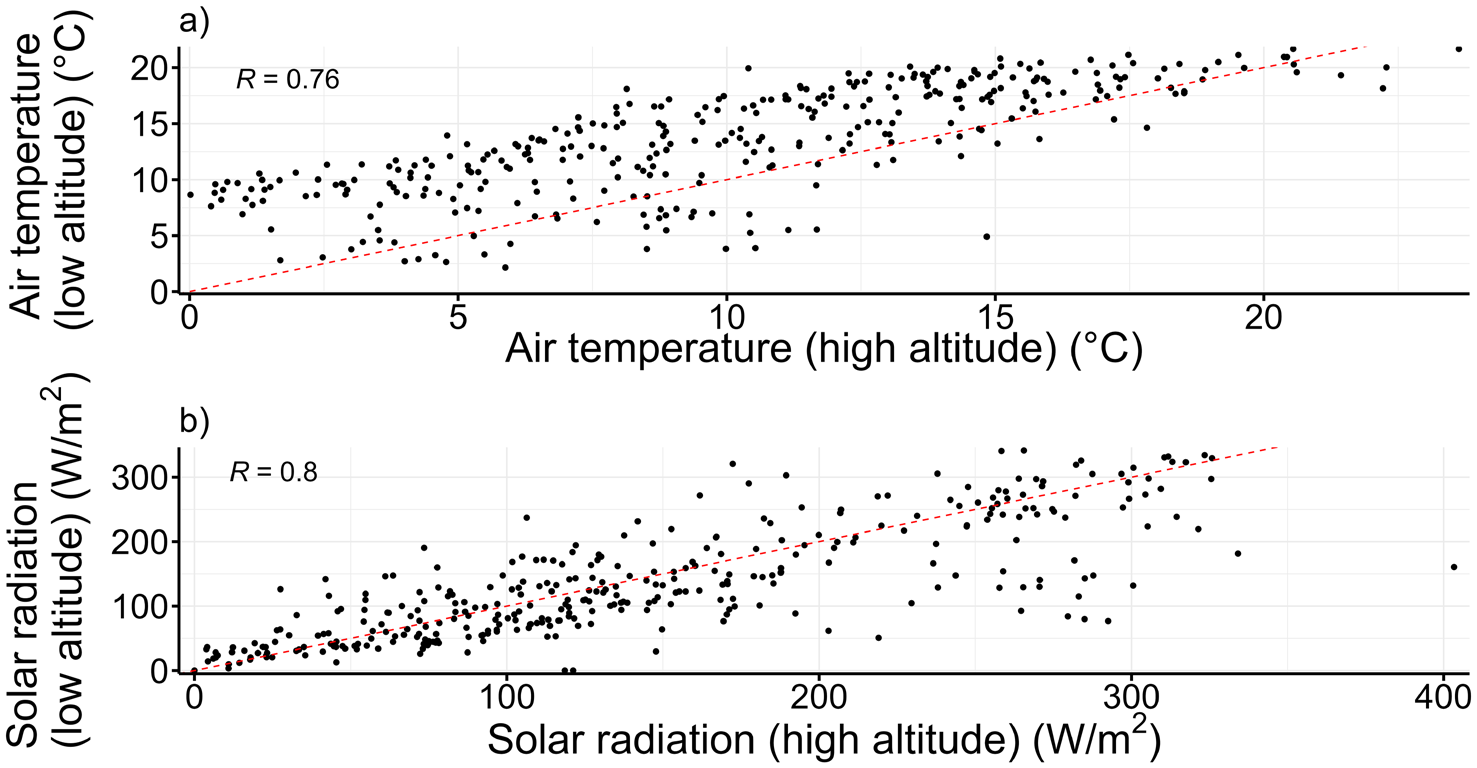
The relationship between (a) air temperature (°C) and (b) solar radiation (W/m^2^) at the high and low altitude site. Air temperature is substantially higher at low altitude whereas solar radiation is very similar between the two sites. Data are daily means for 01/06/2021–31/05/2022, corresponding to the period from when crickets went into boxes up until the median emergence date as an adult. Data were recorded at both sites using Davis instruments, Vantage Pro 2 weather stations (www.davisinstruments.com). The red dotted line indicates the 1:1 relationship. *R* values are the Pearson correlation coefficient.

To manipulate ambient air temperature, boxes from each source population were divided equally between a site at 70 m above sea level (60 boxes) and a site at 1300 m above sea level, 58 km to the southwest (60 boxes). Ambient air temperature was ~4°C lower at the higher altitude site compared to the lower altitude site (daily mean temperature at the high altitude site during the period of our study: 9.6°C, daily mean temperature at the low altitude site: 13.4°C, *t* test, *t* = −9.84, df = 708, *p* ≤ 0.001) (Figure 4a). Mean daily maximum air temperature at the high and low altitude sites was 13.7°C and 18.5°C, respectively. Mean daily minimum air temperature at the high and low altitude sites were 6.0°C and 8.8°C, respectively. Solar radiation can change with altitude (Buckley et al. 2013), but between our sites, there was no systematic difference in solar radiation (Figure 4b) during the period of our study (daily mean radiation at high altitude site: 139.6 W/m^2^, daily mean radiation at low altitude site: 132.8 W/m^2^, *t* test, *t* = 1.05, df = 711, *p* = 0.29). Mean daily maximum solar radiation at the high and low altitude sites was 403.3 and 341.4 W/m^2^, respectively. We hereafter refer to the high and low altitude sites as the low and high air temperature sites, respectively.

To manipulate solar radiation, we partially shaded half of the total number of boxes at each site with a layer of plastic mesh that had a flat profile with 1 mm thick strands, forming rectangular holes of 4 × 5 mm inner size. We used a pyrometer (Davis instruments, Vantage Pro 2 [www.davisinstruments.com]) to determine that this mesh reduced the solar radiation hitting the soil inside the box to about 60% of the value in the unshaded boxes (Figure 3. This is similar to the solar radiative reduction of stratus clouds [~40%–80%]; De Miguel et al. 2011; Matuszko 2012).

*G. campestris* is annual throughout its range, and all adults emerge in the spring following the summer they were laid, at both high and low altitudes. From early April 2022, before adults began to emerge, we started visiting both rearing sites at intervals of a maximum of 11 days (mean: 4 days) to record the date of emergence of new adults and to remove, sex, and weigh them. For crickets showing clear signs of very recent emergence (e.g., still white or observed moulting on the day of the visit), we recorded the visit date as their emergence date. For other newly emerged adults, we estimated their emergence date as the midpoint between the current visit and the previous visit. Some individuals were very difficult to see or to catch during our visits. For these crickets, we estimated their emergence date as the midpoint between the date the first adult was observed at that rearing site and the date we first saw them. In cases where this estimation was not possible, no emergence date was assigned. Adults removed from the rearing boxes were weighed within the following 2 days with a 0.01 g accuracy balance.

## 3 Analysis

We compared the development time (number of days from 1st January to the date of emergence as an adult) and adult mass (in grams) between shaded and unshaded boxes at high and low air temperature sites using fixed‐effects linear models assuming Gaussian errors. Site (air temperature) and shading treatment and their interaction, and altitude of origin were included as predictors, and sex was included as an additional interaction predictor in the adult mass model as females are, on average, larger than males.

Diagnostic plots were checked and showed that model assumptions of normality and homoscedasticity were met. A potential relationship between adult mass and development time, which has been reported previously (Ritz and Köhler 2007), was examined using a Kendall's rank correlation test, but no significant correlation was found (*τ* = −0.057, *z* = −1.11, *p* = 0.27).

All statistical analyses were carried out in R, version 4.3.1 (R Core Team 2023).

## 4 Results

**FIGURE 5:**
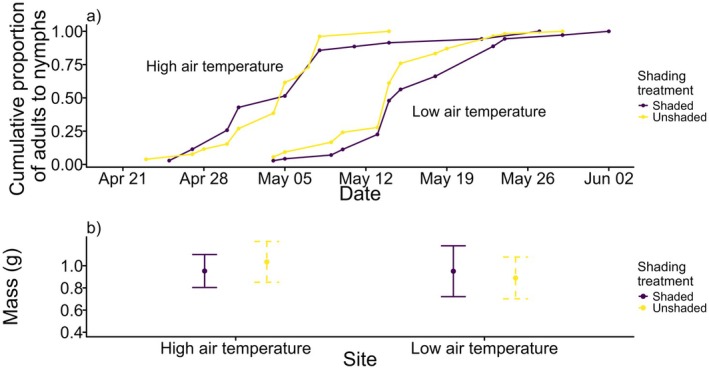
(a) Cricket development (as the cumulative proportion of all adults to nymphs from 23/04/2022 to 02/06/2022) and (b) adult mass (in grams) for high and low air temperature sites for unshaded (yellow lines) and shaded (purple lines) treatments. In (b), points show mean adult mass for the crickets in each treatment, whiskers extend to one standard deviation above and below the mean mass. Sample sizes as follows: High air temperature site: Shaded = 35, unshaded = 26; low air temperature site: Shaded = 71, unshaded = 54.

Development time was strongly affected by air temperature: crickets at the high air temperature site emerged on average 10 days earlier than those in the low air temperature site (*t*_181_ = 6.94, *p* < 0.0001) (Figure 5a). Development time was not significantly affected by whether or not crickets were in shade (est = −1.46 days, *t*_181_ = 0.94, *p* = 0.35). The altitude of origin did not affect development time (*t*_181_ = 0.70, *p* = 0.49). Please see Table 2 for all statistical results.

**TABLE 2:** Results of a Gaussian linear model analyzing predictors of development time (calculated as the number of days after 1st January that a cricket emerged as adult).

Variable	Estimate	SE	*p*
Intercept	122.8	1.47	< 0.0001
Site (low air temperature)	10.04	1.45	< 0.0001
Treatment (shaded)	1.46	1.56	0.35
Altitude of origin (< 120 m)	0.69	0.99	0.49
Site (low air temperature)*treatment (shaded)	1.50	1.90	0.43

*Note:* Total number of observations: 186.

Adult mass was lower at the low air temperature site (Figure 1b) (est = −0.25 g, *t*_177_ = −3.81, *p* < 0.001). Adult mass was also dependent upon an interaction between the air temperature and the availability of solar radiation: at low air temperature, shaded crickets had higher mass (on average + 0.06 g) than unshaded crickets, whereas at high air temperature, shaded crickets had lower mass than unshaded crickets (on average − 0.08 g) (*t*_177_ = 2.29, *p* = 0.02) (Figure 5b). Males had lower mass than females (mean mass of females: 0.97 g, mean mass of males: 0.92 g, est. = −0.19 g, *t*_177_ = −2.46, *p* = 0.156). Altitude of origin did not affect adult mass (*t*_177_ = −0.88, *p* = 0.38). Please see Table 3 for all statistical results.

**TABLE 3:** Results of a Gaussian linear model analyzing predictors of adult mass (in grams).

Variable	Estimate	SE	*p*
Intercept	1.16	0.06	< 0.0001
Site (low air temperature)	−0.26	0.07	< 0.0001
Treatment (shaded)	−0.13	0.08	0.10
Sex (male)	−0.19	0.08	0.02
Altitude of origin (< 120 m)	−0.03	0.03	0.38
Site (low air temperature)*treatment (shaded)	0.21	0.09	0.02
Site (low air temperature)*sex (male)	0.20	0.09	0.04
Treatment (shaded)*sex (male)	0.09	0.10	0.39
Site (low air temperature)*treatment (shaded)*sex (male)	−0.14	0.12	0.26

*Note:* Total number of observations: 186.

We found no significant correlation between adult mass and development time (τ = −0.06, z = −1.11, *p* = 0.27).

## 5 Discussion

### 5.1 Effects of Air Temperature

Nymphs became adult on average 10 days later at lower air temperatures. Development time is an important life history trait and is expected to be under selection (Gregory 2009). Earlier adult maturity has the potential to reduce generation time and reduces the period during which individuals are exposed to predators and other mortality risks prior to reproductive maturity. Hence earlier reproductive maturity tends to increase the probability that an individual passes on its genes before it dies (Gregory 2009). The ‘slow growth high mortality’ hypothesis, for example, suggests that extended development times at lower temperatures may increase the cumulative risk of mortality, as individuals remain vulnerable to predation and other hazards for a longer period (e.g., Benrey and Denno 1997).

In annual species, however, the fixed generation time means that the benefits of earlier maturity for parents are weighed against the requirement for their first offspring to then live longer to remain in sync with the annual cycle. Little is known about how phenological shifts or changes in photoperiod affect the suitability of the environment for the growth of crickets, but it is inevitable that seasonal effects will create an optimal period for egg laying and nymphal development, which means that very early or late adult maturity is likely to be selected against; and so overall, we would expect stabilizing selection on development time. We previously identified a positive relationship between early emergence and lifespan in *G. campestris* males (Rodríguez‐Muñoz et al. 2019). However, a subsequent analysis measuring selection as the number of adult offspring produced in the following year (Rodríguez‐Muñoz et al. 2025) reveals that across 8 years, selection on development time tends to be neutral.

Another study (Ritz and Köhler 2007) reported earlier emergence in larger nymphs in a wild population of *G. campestris* . However, this assertion was based on a post hoc analysis showing that the mean size (pronotum width) of newly marked males decreased over a 10‐day period at the beginning of the breeding season. The approach of observing a trend over part of a time series and analyzing that trend in isolation from the subsequent increase in average size carries a high risk of a Type 1 error. Our analysis, in which we can measure the correlation between body mass and development time within individuals, fails to find such a relationship.

Air temperature declines with altitude because the air is less dense. In many ectotherms, individuals grow more slowly but are larger as adults in colder environments (Atkinson (1994, 1995)), an effect known as the temperature‐size rule (Atkinson 1996). However, in this annual species, we found that despite developing more slowly, nymphs matured at a lower adult mass in the lower air temperature treatment. This may be because temperatures at the high air temperature site were not high enough to compromise growth efficiency, or more generally, because of the complex relationship between temperature and body size (Angilletta and Dunham 2003). An earlier analysis of a single year at our low altitude site found that larger *G. campestris* adults had significantly higher lifetime reproductive success (Rodríguez‐Muñoz et al. 2010). However, subsequent analysis across 8 years indicates that this is not a general pattern, and that there is no consistent directional selection in favor of larger body size (Rodríguez‐Muñoz et al. 2025). Other authors have reported a trade‐off whereby lower mass becomes more advantageous at lower temperatures (Olalla‐Tárraga and Rodríguez 2007) since heating rates are determined by body mass (as body mass decreases, body surface area gets proportionally larger, which contributes to increased rates of heat gain, which would be beneficial in a colder environment). Although a possible explanation for the effect we observed, we would not expect the difference between small and large crickets to be biologically meaningful at that level—the thermal inertia of the smallest and largest crickets would be both low and similar. We do note that the difference in mass between the high and low air temperature site, although significant, was relatively small (range in adult mass high air temperature: 0.62–1.52 g, low air temperature: 0.41—1.46 g) compared to the large effect on development time (range in development time high air temperature: 112–146 days, low air temperature: 123–152 days), so it does appear that crickets are optimizing adult mass at the expense of taking longer to develop.

### 5.2 Effects of Solar Radiation

Our shading treatment imposed a significant reduction in solar radiation on our crickets, so we were expecting a large effect on the key life history traits of development rate and adult mass. However, crickets developed at similar rates in the shaded and unshaded treatments, suggesting that they can compensate for suboptimal radiative environments. It is likely that shaded crickets compensated for reduced solar radiation by increasing the amount of time they spent sun basking (Gardner, Li, et al. 2024). This assertion is supported by the observation that *G. campestris* adults spend more time basking when under partial shade (Gardner, Li, et al. 2024); if nymphs behave in the same way, then this would drive the patterns we observe.

Assuming that our nymphs are indeed compensating for partial shade by extending their basking time, this capacity to compensate means that unshaded crickets at high altitude are forgoing the opportunity to bask more, that is, there is sun available that they are not using. Moving in and out of the shade of the burrow only requires very small amounts of energy, so it does not seem likely that crickets abstain from basking because of movement costs. Predation is a likely driver of basking avoidance (Huey and Slatkin 1976), particularly early in the season when the sward height is very short, and so predation risk from birds is likely to be high. Pitt (1999) for example, found that grasshoppers utilize different microhabitats (positions in vegetation) to balance the trade‐off between reducing mortality from predators and experiencing greater food availability and warmer conditions. Several studies have also found that solar UVB radiation can negatively impact development, survival, and reproduction in insects (Mazza et al. 1999; Potter and Woods 2013). When shaded, the benefits of basking (i.e., to maintain development time) seemingly outweigh these potential costs. In our study, natural predation by birds and mammals was prevented because the boxes containing the crickets were covered with a thin mesh. This meant that increased basking by shaded crickets was not penalized by potentially higher predation rates, so if there are interactions between shading, basking behavior, and predation mortality (e.g., Battisti et al. 2013) we would not expect to observe it directly in our study.

Shading had a negative effect on adult mass at the high air temperature site (mean difference − 0.08 g), but a positive effect on adult mass at the low air temperature site (mean difference + 0.06 g). Battisti et al. (2013) report a similar effect of shading on first‐instar colonies of *Thaumetopoea pinivora*; shading did not affect the developmental rate but resulted in smaller body size. The authors suggest that the greater size achieved for the same development time in unshaded colonies could increase their capacity to resist predation. Predation risk does not seem to be related to size in field crickets—the majority of predation events that we observe are by large predators such as robins and shrews (Rodríguez‐Muñoz et al. 2011) which can handle the largest crickets. Slower growth rates, which lead to larger body mass, may enhance an organism's ability to conserve heat (Meiri 2011). However, as body mass increases, the rate of heat gain tends to decline. Given that all crickets are small, their thermal inertia should always be very small, making it unlikely that any effect of mass is related to differences in thermal inertia.

### 5.3 Local Adaptation

We found no evidence for local adaptation influencing responses to air temperature or shading in post‐diapause growth. This suggests that crickets from high altitude genetic populations are not genetically adapted to anticipate and respond to cooler conditions. This finding contrasts with our earlier observation of an interaction between genetic background and rearing temperature affecting the growth rate of very small nymphs in the period immediately post‐hatching, which, like the crickets in this study, were provided with ad lib food. Our earlier observation suggests that local genetic adaptation to altitude can occur in the populations that we studied, and a RADseq genetic study found genetic differentiation between these populations. Therefore, the lack of any evidence of such adaptation in this study indicates that there is not very strong selection for differences in growth rate, adult mass, or interactions between these traits and air temperature and solar radiation availability in post‐diapause nymphs. This finding is somewhat unexpected because the much shorter breeding seasons at high altitude might be expected to favor more rapid growth when heat is available. However, precise predictions are hard to make because a shorter breeding season also means that there is a longer pre‐adult phase, which provides more time for growth before mates are available.

## 6 Conclusions

Although we report effects of ambient temperature and shading on life history traits in nymphs, how these factors affect lifetime fitness (in term of the probability that an individual of a given phenotype will contribute to subsequent generations (Lailvaux and Husak 2014)) remains unknown. It would be extremely difficult to track individual crickets from nymphs to adults in a natural context. However, in future studies, we could analyze how air temperature and access to solar radiation affect fitness or fitness‐related traits in adult crickets. An interesting mechanistic approach to this would be to relate individual body temperature and fitness. There are likely complex trade‐offs and strategies to optimize fitness in a particular environment (Lahondère 2023).

Better understanding of the possible fitness consequences of thermal environments for ectotherms, including possible trade‐offs of increased basking activity under different temperature regimes (e.g., Ma et al. 2018), will help to uncover the drivers governing their distributions (Larsson 2002). This will benefit numerous fields, including ecology and biogeography, with applications in the assessment of pest risk (e.g., Mezei et al. 2019) and the possible consequences of climate change. Given that temperature and solar radiation (through changes to cloud cover) are important components of climate change (although future trends in cloudiness and radiation are complex to predict) (IPCC 2013; Cox et al. 2020), understanding how warmer temperatures and altered basking opportunities (due to changes in cloud cover) might interact and impact ectotherms becomes crucial. Our results suggest that developing crickets forgo some opportunities to gain energy from the sun, because if shaded they can compensate to maintain the development rate. Our observation that the direction of the effect of shade on adult mass depends on air temperature means that changes in cloud cover may impact insects differently in warmer and cooler parts of their range.

## Author Contributions

**Alexandra S. Gardner:** formal analysis (equal), investigation (equal), methodology (equal), visualization (equal), writing – original draft (equal), writing – review and editing (equal). **Ilya M. D. Maclean:** conceptualization (equal), formal analysis (equal), funding acquisition (equal), investigation (equal), methodology (equal), project administration (equal), resources (equal), software (equal), supervision (equal), writing – original draft (equal), writing – review and editing (equal). **Rolando Rodríguez‐Muñoz:** conceptualization (equal), data curation (equal), formal analysis (equal), investigation (equal), methodology (equal), project administration (equal), resources (equal), supervision (equal), writing – review and editing (equal). **Alfredo F. Ojanguren:** investigation (equal), methodology (equal), writing – review and editing (equal). **Tom Tregenza:** conceptualization (equal), data curation (equal), formal analysis (equal), funding acquisition (equal), investigation (equal), methodology (equal), project administration (equal), resources (equal), software (equal), supervision (equal), writing – original draft (equal), writing – review and editing (equal).

## Ethics Statement

This study has been approved by the University of Exeter's Research Ethics Panel, approval number 513752. All crickets used in the study will live out their natural lives in the wild.

## Conflicts of Interest

The authors declare no conflicts of interest.

## Data Availability

The data used in the analysis are deposited in Zenodo: https://zenodo.org/records/14679397.
